# Spatial Epidemiological Approach to Tuberculosis Treatment Outcomes in a Tertiary-Level Hospital: A Retrospective Analysis

**DOI:** 10.3390/tropicalmed11020057

**Published:** 2026-02-18

**Authors:** Luis Eduardo Del Moral Trinidad, Gilberto Silva Bañuelos, Esteban Gonzalez-Diaz, Melva Guadalupe Herrera Godina

**Affiliations:** 1 Doctorado en Ciencias de la Salud Pública, Centro Universitario de Ciencias de la Salud, Universidad de Guadalajara, Guadalajara 44430, Mexico; l.eduardo.trinidad@outlook.com; 2 Departamento de Salud Pública, Centro Universitario de Ciencias de la Salud, Universidad de Guadalajara, Guadalajara 44430, Mexico; gil.saludpublica@gmail.com; 3 Unidad de Medicina Preventiva y Vigilancia Epidemiológica, Hospital Civil de Guadalajara “Fray Antonio Alcalde”, Guadalajara 44280, Mexico; esteban.gdiaz@academicos.udg.mx; 4 Instituto de Patología Infecciosa y Experimental “Francisco Ruiz Sánchez”, Centro Universitario de Ciencias de la Salud, Universidad de Guadalajara, Guadalajara 44430, Mexico

**Keywords:** tuberculosis, treatment outcomes, spatial epidemiology, Mexico

## Abstract

Tuberculosis (TB) remains a persistent public health challenge in Mexico, particularly in large urban settings marked by social heterogeneity. We conducted a retrospective cohort study of patients diagnosed with tuberculosis and treated at a tertiary-level hospital in Guadalajara, Mexico, between 2020 and 2023. Unfavorable treatment outcomes were defined as treatment failure, loss to follow-up, or death. Multivariable logistic regression was used to identify factors independently associated with unfavorable outcomes. Spatial analyses, including Kernel Density Estimation, Global Moran’s I, Local Indicators of Spatial Association (LISA), and Getis–Ord Gi*, were applied to explore the geographic distribution of unfavorable outcomes. Unfavorable tuberculosis treatment outcomes among patients treated at a tertiary-level hospital were not randomly distributed in space. Spatial epidemiological methods provided complementary, exploratory insights beyond individual-level clinical factors, highlighting geographic patterns that may inform place-sensitive public health interventions and strengthen routine tuberculosis surveillance, without implying causal inference.

## 1. Introduction

Tuberculosis remains a significant global health challenge, accounting for approximately 9 million new cases and 1.5 million fatalities annually on a worldwide scale. Mexico faces this challenge as well, with an incidence rate of 19 cases per 100,000 population and a mortality rate of 1.68 per 100,000 in 2019, highlighting the critical need for robust strategies to combat this persistent public health issue [1]. The emergence of the COVID-19 pandemic has presented additional challenges to TB control initiatives, leading to a significant global reduction in diagnoses and the commencement of treatment [2].

Effective management of the tuberculosis epidemic hinges on robust pharmacotherapy. However, outcomes are varied, with some patients experiencing treatment failure, loss to follow-up or mortality. Factors influencing these outcomes are multifaceted and include socio-economic status, healthcare accessibility, treatment adherence, and the presence of comorbidities [3,4,5]. These factors are not uniformly distributed across populations or territories, and there is increasing recognition that spatial context plays a significant role in shaping health outcomes [6].

Geographic information systems (GIS) and spatial epidemiology have emerged as critical tools in understanding the distribution of diseases and health outcomes. These methods allow researchers and public health authorities to identify clusters, assess risk environments, and detect geographic inequalities that may be invisible in conventional analysis [7]. In infectious diseases, spatial patterns often reflect underlying structural disparities such as limited access to healthcare, urban overcrowding, or environmental exposures [8].

In the case of TB, spatial analysis is particularly valuable because the disease is influenced by a combination of biological, social, and structural determinants that vary across territory. For example, overcrowded neighborhoods, low-income areas, and zones with poor access to health services have been associated with increased transmission and worse treatment outcomes. By mapping these patterns, health authorities can prioritize interventions and allocate resources more effectively, particularly in high-burden urban settings [9,10].

Despite this potential, few studies in Mexico have incorporated spatial analysis into the evaluation of TB outcomes. This lack of evidence limits the ability to understand how geographic disparities may have influenced treatment success or failure during a time of substantial health system disruption. Incorporating a geospatial perspective can help identify areas where structural vulnerabilities intensified during the pandemic, offering valuable insights for targeted public health strategies [6].

Geographic disparities in health outcomes often reflect differences in access to care, local infrastructure, and broader social determinants of health. In the context of TB treatment, spatial barriers and heterogeneity in the availability and quality of healthcare services may significantly influence therapeutic outcomes [2,11].

Despite the evidence describing the clinical and social determinants of tuberculosis outcomes, limited attention has been given to their spatial distribution, particularly in urban settings with a high TB burden. In Mexico, few studies have incorporated a geospatial perspective to evaluate the effect of place-based inequalities on TB outcomes in tertiary-level institutions. Accordingly, this study aims to analyze the spatial distribution of tuberculosis treatment outcomes and to examine their relationship with individual- and context-level factors in patients diagnosed at a tertiary-level referral hospital from 2020 to 2023.

## 2. Materials and Methods

### 2.1. Study Design and Setting

A descriptive observational study was conducted to evaluate tuberculosis treatment outcomes using spatial analysis techniques to map the distribution of treatment results and assess their association with sociodemographic and clinical variables at the Hospital Civil de Guadalajara “Fray Antonio Alcalde” in Jalisco, Mexico, from January 2020 to December 2023. All patients diagnosed during the study period were included, and their demographic, clinical, and treatment data were collected for subsequent analysis.

### 2.2. Study Population and Sample Size

All patients diagnosed during the study period were recruited, and the initial cohort comprised 943 patients. Following the exclusion of 49 individuals with any form of drug resistance, 33 with incomplete data, and 66 who did not commence treatment, a final sample of 795 patients was included in the analysis.

Patients with drug-resistant tuberculosis were excluded because they follow distinct diagnostic, therapeutic, and follow-up protocols managed by specialized referral committees, which substantially differ from those used for drug-susceptible tuberculosis. Including these patients would have introduced clinical and programmatic heterogeneity, potentially biasing the estimation of treatment outcomes and limiting comparability across cases.

Additionally, patients who did not initiate treatment were excluded because treatment outcomes could not be ascertained in the absence of therapeutic follow-up, precluding their inclusion in outcome-based regression and spatial analyses.

### 2.3. Study Procedure

A retrospective review of patients with confirmed tuberculosis at the hospital unit was performed. Sociodemographic and clinical data were gathered using a data collection form and subsequently transferred to an electronic spreadsheet. Patients diagnosed in our facility are referred to their local community health centers for treatment initiation. Consequently, their treatment outcomes were tracked through the national platform, where such data is systematically recorded.

Following the collection of patient residential addresses, geographic coordinates were obtained using Google Maps and subsequently projected to UTM Zone 13N for spatial analyses conducted in ArcGIS Pro. Of the 828 registered tuberculosis cases, 795 (96.0%) were successfully geocoded at the residential address level.

A total of 33 cases (4.0%) were excluded from spatial analyses due to incomplete, ambiguous, or insufficient address information that precluded reliable georeferencing. No spatial interpolation was applied for these records.

All successfully geocoded locations were visually inspected to ensure consistency with the reported municipality and Basic Geostatistical Area (AGEB). Formal quantification of positional uncertainty was not performed; however, geocoding accuracy was considered adequate for AGEB-level aggregation and spatial pattern analysis.

### 2.4. Study Variables

The dependent variable was the treatment outcome, categorized as successful and unfavorable. The definitions used are those mentioned by the World Health Organization [12]. Successful outcomes included treatment completed and cured, while unfavorable outcomes comprised treatment failure, loss to follow-up, and death, regardless of whether the death was directly attributable to TB. Specifically, treatment failure was defined as a patient remaining smear or culture-positive at month 5 or later during treatment. Loss to follow-up referred to patients whose treatment was interrupted for two consecutive months or more. Death included any patient who died for any reason before or during the course of treatment.

All classifications were made based on final case closure as recorded in the national electronic TB surveillance system and verified by clinical records. The unfavorable outcome variable was coded dichotomously for the purposes of statistical analysis.

The independent variables were sex, age, education level divided into grades, and occupation, with responses grouped into different categories to reduce the number of possibilities. The ‘other unique professions’ category was included for informal jobs that encompass a wide range, such as hairstylists, cruise workers, and others. Additional variables included year of diagnosis, active smoking, active alcoholism, injection drug use at the time of diagnosis, immunosuppression conditions, the HIV/TB co-infection, history of BCG vaccination, and disease location. Immunosuppression not related to HIV infection included conditions such as chronic kidney disease, malignancies, solid organ or hematologic transplantation, long-term systemic corticosteroid use, immunosuppressive or biologic therapies, and other clinically documented causes of impaired immune function.

Geographic coordinates derived from patients’ residential addresses were included as contextual variables for spatial analysis.

### 2.5. Data Analysis

The patient data was extracted from electronic medical records and entered into an Excel spreadsheet. Then the data was exported into statistical packages for social sciences version 24 (SPSS V24) for analysis. Descriptive statistics were used to describe participants’ characteristics, treatment outcomes and year trends.

A descriptive statistical analysis was conducted on the sociodemographic and clinical characteristics of patients with tuberculosis. Categorical variables were summarized using absolute frequencies and percentages, while continuous variables were described using means and standard deviations or medians and interquartile ranges, depending on their distribution, which was assessed using the Shapiro–Wilk test.

A binary variable was created for treatment outcomes, classifying cases as successful (cured or treatment completed) or unfavorable (treatment failure, death, lost to follow-up). The distribution of treatment outcomes was explored by year of diagnosis and by clinical and sociodemographic categories.

Subsequently, bivariate analyses were performed to identify associations between treatment outcome and independent variables. The chi-square test or Fisher’s exact test was used for categorical variables, and Student’s *t*-test or the Mann–Whitney U test was applied for continuous variables, as appropriate.

To identify factors associated with unfavorable outcomes (defined as treatment failure, loss to follow-up, or death, whether TB-related or unrelated), we performed univariate and multivariate logistic regression analyses. Crude odds ratios (ORs) and their 95% confidence intervals (CIs) were first calculated for each independent variable using simple logistic models. Subsequently, a multivariate logistic regression model was constructed, including variables that were statistically significant in the bivariate analysis or considered clinically relevant based on the literature. Adjusted odds ratios (AORs) and 95% CIs were reported.

### 2.6. Spatial Analysis

The spatial unit used in this analysis was the Área Geoestadística Básica (AGEB), which is the smallest official geographic division used by the Mexican National Institute of Statistics and Geography (INEGI) [13,14]. Individual-level geocoded residential addresses were first aggregated to the AGEB level, which represents the smallest census unit available for population-based spatial analyses in Mexico. Each TB case was assigned to its corresponding AGEB based on residential location at the time of diagnosis.

For descriptive mapping, case counts and proportions of unfavorable treatment outcomes (treatment failure, loss to follow-up, or death) were calculated for each AGEB. Choropleth maps were generated to visualize the spatial distribution of cases and outcomes. For these maps, the numerator corresponded to the number of individuals with a given outcome within each AGEB, while the denominator corresponded to the total number of TB cases recorded in that AGEB during the study period. No population-standardized incidence rates were calculated, as the analysis focused on the spatial distribution of TB outcomes among diagnosed cases rather than population-level risk.

To explore spatial concentration patterns, kernel density estimation (KDE) was applied to point-level case data using a fixed bandwidth to identify areas with higher densities of unfavorable outcomes. Kernel density maps were generated using geographic coordinates of individual residences, allowing visualization of spatial clustering independent of administrative boundaries.

Spatial clustering of tuberculosis cases was evaluated using the Local Indicators of Spatial Association (LISA), specifically the Local Moran’s I (Anselin) statistic, computed annually for the years 2020 to 2023. The analysis was performed at the municipality level within the Guadalajara Metropolitan Area, based on the number of confirmed TB cases per municipality. Crude case counts were used for the calculation of spatial autocorrelation, and a false discovery rate (FDR) correction was applied to adjust for multiple comparisons. The classification of spatial outliers and clusters included high–high (HH), low–low (LL), high–low (HL), and low–high (LH) patterns, although only statistically significant clusters are shown [15]. No smoothing procedures were applied to the data beyond cartographic visualization.

Spatial autocorrelation was assessed using Global Moran’s I, applied to AGEB-level proportions of unfavorable outcomes [15]. This analysis evaluated whether the observed spatial pattern differed from a random spatial distribution. Subsequently, Getis–Ord Gi* statistics were computed to identify statistically significant hot spots and cold spots of unfavorable outcomes. These analyses were based on contiguity-based spatial weights and used standardized z-scores to identify clusters at the 95% and 99% confidence levels. All spatial analyses were conducted using ArcGIS Pro, version 3.3 (Esri Inc., Redlands, CA, USA, 2024)

Spatial weights matrices for Global Moran’s I, LISA, and Getis–Ord Gi* analyses were constructed using a first-order contiguity-based approach (queen contiguity), which is appropriate for areal units such as AGEBs and allows each unit to be influenced by all adjacent neighbors sharing either a border or a vertex [15]. This approach was selected to reflect local spatial dependence while avoiding arbitrary distance thresholds.

Kernel Density Estimation was performed using a Gaussian kernel function with a fixed bandwidth selected according to ArcGIS Pro default optimization parameters, expressed in meters, to identify areas of higher concentration of unfavorable treatment outcomes without administrative boundary constraints.

Getis–Ord Gi* hot spot analyses were conducted using the same contiguity-based spatial weights, with statistical significance assessed through 999 permutations and standardized z-scores, identifying hot spots and cold spots at the 95% and 99% confidence levels. A false discovery rate (FDR) correction was applied to Local Moran’s I statistics to adjust for multiple comparisons.

### 2.7. Ethics

Given the retrospective nature of the study, informed consent was not required. A full study protocol, which included an informed consent waiver form, was prepared and approved by the institutional bioethics committee (registration number CEI 53/23 approved on 20 February 2025). To ensure data confidentiality, sensitive data collection was omitted, and alphanumeric identifiers were used to prevent identification. To protect participant confidentiality during the geocoding process, residential addresses were used solely to obtain geographic coordinates and were not retained in the analytical dataset. All spatial analyses were conducted using anonymized data, and results were presented at aggregated levels (AGEB or municipality), preventing individual identification. Access to geocoded data was restricted to the research team. This project adheres to the principles of the Declaration of Helsinki and guidelines established by the hospital’s ethics committee.

## 3. Results

### 3.1. Population Characteristics

Table 1 summarizes the sociodemographic and clinical characteristics of the study population stratified by treatment outcome. The median age was slightly higher in the unfavorable outcome group compared to those with successful outcomes, though the difference was not statistically significant (41 vs. 39 years, *p* = 0.446). Male patients represented a significantly higher proportion of those with unfavorable outcomes (*p* = 0.008). Educational attainment was also associated with outcomes; patients with no formal education or only primary education had a greater proportion of unfavorable outcomes compared to those with higher education levels (*p* = 0.023).

Occupational category was significantly related to treatment outcomes (*p* < 0.001), with unfavorable outcomes more common among the unemployed. Pulmonary TB was significantly associated with unfavorable outcomes (*p* = 0.004). Immunosuppression unrelated to HIV was more frequent among those with unfavorable outcomes (*p* = 0.034). Unfavorable outcomes were also higher in 2021 and 2022 compared to 2023 (*p* < 0.001).

### 3.2. Factors Associated with Unfavorable Treatment Outcomes: Crude and Adjusted Odds Ratios

In the unadjusted analyses, male sex (OR = 1.68; 95% CI: 1.05–2.70), lack of formal education (OR = 1.70; 95% CI: 1.02–2.84), unemployment (OR = 1.74; 95% CI: 1.17–2.58), pulmonary TB (OR = 1.75; 95% CI: 1.13–2.69), and year of diagnosis (2021 and 2022) were significantly associated with unfavorable outcomes. In contrast, TB diagnosis in 2023 was associated with a lower risk (OR = 0.03; 95% CI: 0.00–0.23).

In the multivariate model, only pulmonary TB (AOR = 2.05; 95% CI: 1.27–3.32), diagnosis in 2021 (AOR = 2.23; 95% CI: 1.14–4.36), in 2022 (AOR = 2.27; 95% CI: 1.16–4.30), and in 2023 (AOR = 0.03; 95% CI: 0.01–0.20) remained statistically significant predictors (Table 2).

### 3.3. Spatial Distribution

**Figure 1:**
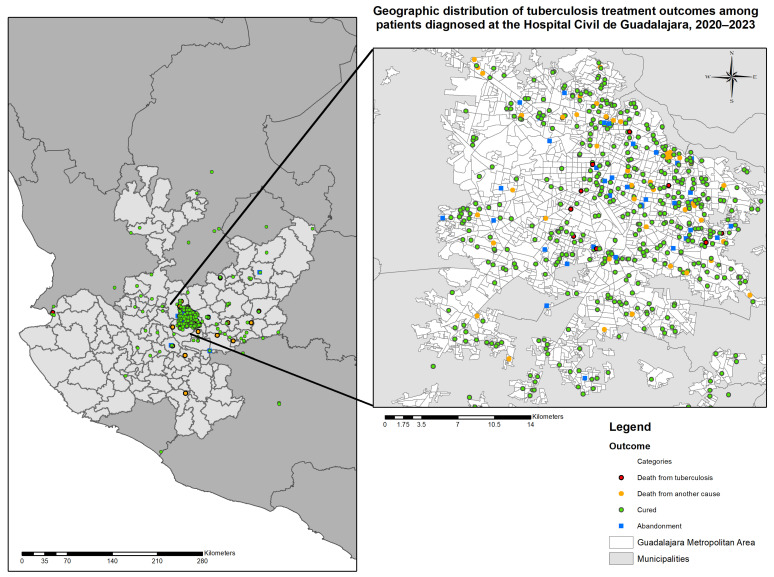
Geographic distribution of tuberculosis treatment outcomes in patients diagnosed at the Hospital Civil de Guadalajara “Fray Antonio Alcalde”, from January 2020 to December 2023. Each dot represents an individual case and is color-coded according to treatment outcome, based on raw, non-aggregated point-level data from patients’ residential addresses. The left panel shows the regional distribution across the state of Jalisco, while the right panel focuses on the Guadalajara Metropolitan Area.

The spatial distribution of TB treatment outcomes was mapped across the state of Jalisco and the Guadalajara Metropolitan Area. Most patients were concentrated in the central metropolitan area, with scattered cases in peripheral municipalities. A dot map was constructed to visualize individual outcomes geographically, showing a higher density of unfavorable outcomes (treatment failure, loss to follow-up, death) in specific urban zones (Figure 1).

**Figure 2:**
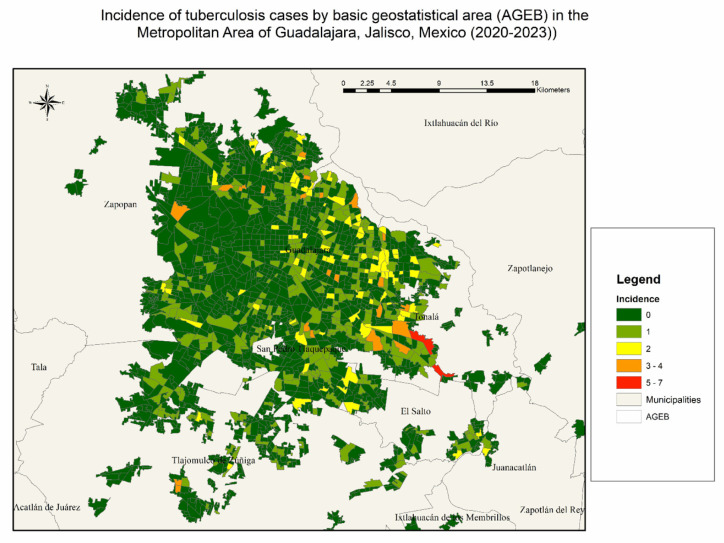
Incidence of tuberculosis cases by basic geostatistical area (AGEB) in the Metropolitan Area of Guadalajara, Jalisco, Mexico (2020–2023). Maps were created using crude case counts per AGEB, aggregated across the 4-year period. Incidence levels were classified into six categories (0 to 7+ cases) and visualized using a color gradient from green (low) to red (high).

The map demonstrates considerable geographic variation, with areas of higher incidence concentrated in the southeastern region, particularly in parts of Tonalá and Tlaquepaque. These high-incidence clusters contrast with extensive areas reporting zero or very low case counts, primarily located in the western zones such as Zapopan (Figure 2).

**Figure 3:**
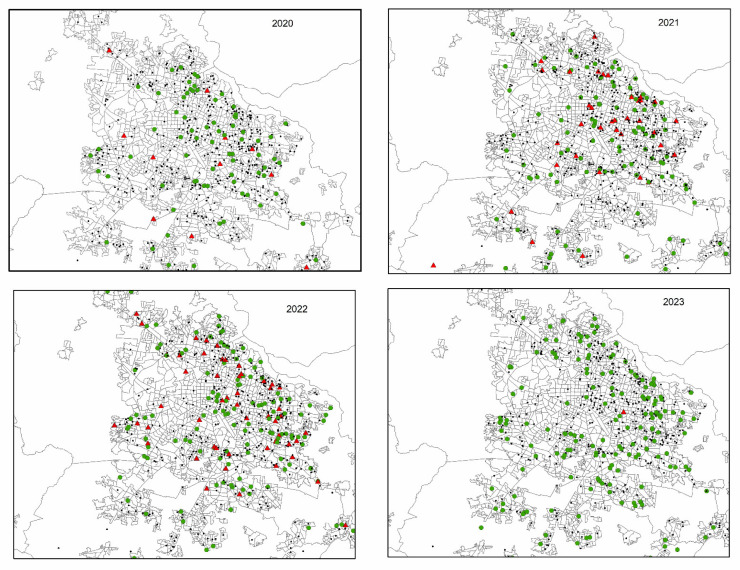
Annual distribution of tuberculosis cases and treatment outcomes in the Guadalajara Metropolitan Area, Jalisco, Mexico (2020–2023). Each panel shows raw point data representing the residential address of patients diagnosed with tuberculosis in the respective year. Green circles represent favorable outcomes (cure or treatment completion), and red triangles indicate unfavorable outcomes (failure, loss to follow-up, or death). The symbols were georeferenced using patients’ reported residential addresses and mapped according to their location within Basic Geostatistical Areas (*Áreas Geoestadísticas Básicas*, AGEBs).

Throughout the study period, a consistent concentration of cases was observed in central and eastern areas of the city. In 2021 and 2022, there was a noticeable increase in the number and geographic spread of unfavorable outcomes, especially in peripheral zones. By 2023, favorable outcomes predominated, and spatial dispersion appeared more evenly distributed across urban and peri-urban areas (Figure 3).

**Figure 4:**
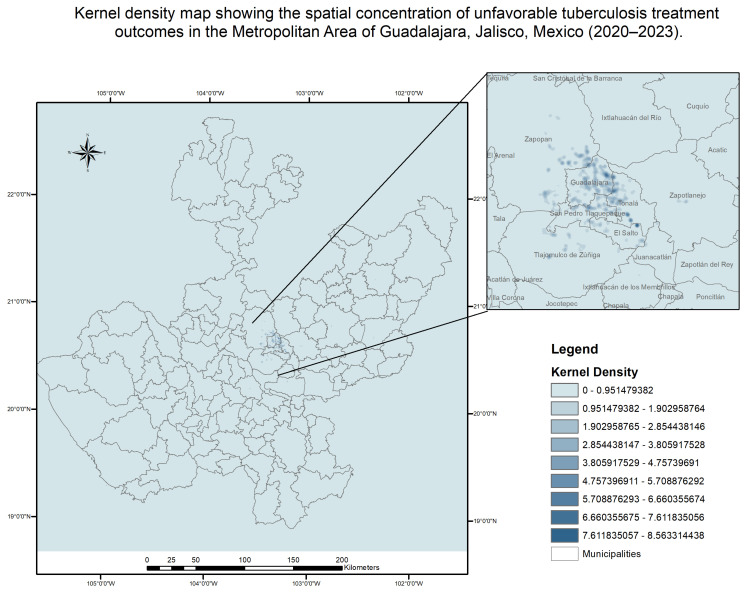
Kernel density map of unfavorable tuberculosis treatment outcomes in the Guadalajara Metropolitan Area, Jalisco, Mexico (2020–2023). The map was generated using kernel density estimation (KDE) based on point data of individual cases with unfavorable outcomes.

Kernel density analysis reveals the concentration of unfavorable outcome distribution. A notable concentration of cases with unfavorable results is observed in the center of the metropolitan area, particularly in the northeast and southeast zones (Figure 4).

**Figure 5:**
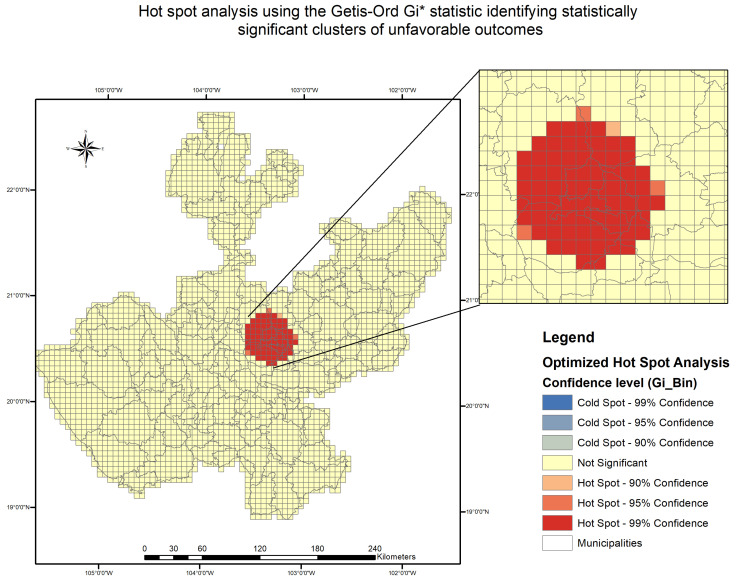
Hot spot analysis of unfavorable tuberculosis treatment outcomes in the Guadalajara Metropolitan Area (2020–2023), using the Getis–Ord Gi* statistic. The map identifies statistically significant spatial clusters of high (hot spots) and low (cold spots) concentrations of unfavorable outcomes, with 99% confidence intervals. Analysis was conducted using raw counts of unfavorable outcomes per AGEB.

Figure 5 displays the results of a hot spot analysis using the Getis–Ord Gi* statistic. A statistically significant hot spot at the 99% confidence level was detected in central Guadalajara, indicating a spatial cluster of high rates of unfavorable outcomes. No cold spots were observed.

These patterns were confirmed by the global spatial autocorrelation analysis (Global Moran’s I), which yielded a Moran’s I index of 0–24, with a z-score of 2.25 and a *p*-value of 0.024, indicating significant spatial clustering of unfavorable treatment outcomes across the study region.

**Figure 6:**
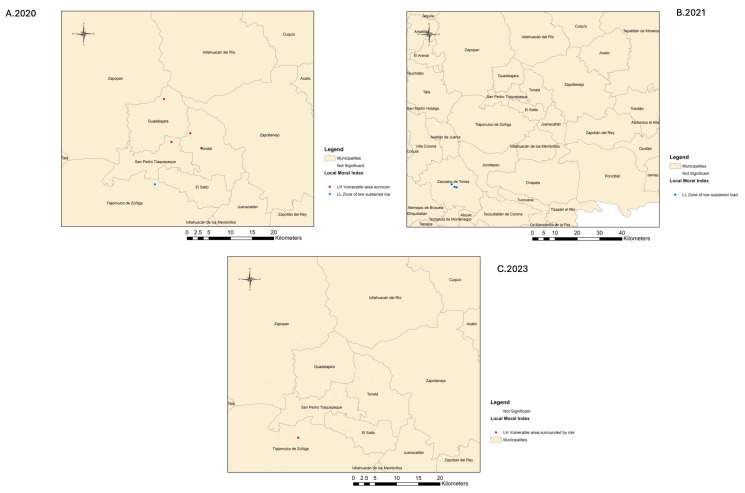
Spatial clusters and outliers of tuberculosis cases in the Guadalajara Metropolitan Area, Jalisco, Mexico, for the years 2020 (**A**), 2021 (**B**), and 2023 (**C**), based on the Local Moran’s I statistic (Anselin) with false discovery rate (FDR) correction. LH = low–high (vulnerable areas surrounded by higher-risk neighbors); LL = low–low (areas of low sustained burden). Only statistically significant clusters are shown. 2022 was not computed due to the absence of statistically significant spatial clusters.

The spatial distribution of tuberculosis cases showed temporal variation in clustering patterns across the years analyzed. In 2020, several statistically significant low–high (LH) clusters were detected in central municipalities such as Guadalajara, Tlaquepaque, and Tonalá, indicating vulnerable areas surrounded by higher-burden neighbors. One low–low (LL) cluster was also detected in the southern municipality of Tlajomulco. In 2021, a single LL cluster was identified in Zacoalco de Torres, outside the central urban area, with no high-burden clusters detected. In contrast, 2023 revealed only one LH cluster in Tlajomulco de Zúñiga. The absence of significant clusters in 2022 suggests a potential disruption in spatial patterns, likely influenced by the COVID-19 pandemic. Taken together, these results indicate a dynamic evolution of spatial TB burden, with a transient increase in clustering during the early pandemic period (2020), followed by spatial dispersion and fewer detectable clusters in subsequent years (Figure 6).

## 4. Discussion

In this study, we described the treatment outcomes of tuberculosis in a cohort of patients diagnosed at a tertiary-level hospital in Western Mexico, incorporating a spatial epidemiological approach to analyze the geographical distribution of unfavorable outcomes and identify potential patterns of concentration or territorial clustering. While some sociodemographic and clinical characteristics showed differences in the descriptive analysis, the multivariable model identified pulmonary tuberculosis and year of diagnosis as the main independent predictors of unfavorable treatment outcomes. Importantly, the spatial analyses revealed non-random geographic clustering of unfavorable outcomes that was not fully explained by individual-level clinical variables, highlighting the potential relevance of contextual and territorial factors in shaping treatment success.

The descriptive patterns observed in this cohort are consistent with previous studies reporting higher proportions of unfavorable outcomes among men, older individuals, and patients with comorbidities such as diabetes mellitus, HIV infection, and other immunosuppressive conditions [5,16,17]. However, these factors did not remain independently associated with unfavorable outcomes in the multivariable model. Rather than indicating isolated individual predictors, these descriptive patterns provide contextual insight into vulnerability profiles that may interact with territorial characteristics, reinforcing the need to interpret tuberculosis treatment outcomes within an integrated clinical–spatial framework [18].

A notable contribution of this study is the demonstration that spatial clustering of unfavorable tuberculosis treatment outcomes persists even when few individual-level variables remain significant in multivariable models. The identification of statistically significant clusters and hot spots through Global Moran’s I, LISA, and Getis–Ord Gi* analyses indicates that geographic space itself captures dimensions of risk not fully represented in routinely collected clinical data [19]. These findings support the interpretation of spatial analysis as a complementary tool to regression modeling, capable of highlighting geographic patterns that may reflect the influence of unmeasured contextual factors—such as access to health services, transportation barriers, social marginalization, or local health system characteristics—that can affect continuity of care and treatment adherence [6].

From a conceptual standpoint, the spatial analyses in this study were designed as exploratory tools to identify geographic concentrations of unfavorable tuberculosis treatment outcomes, rather than to establish causal relationships. Observed spatial clustering should therefore be interpreted as a signal of spatially structured processes that may operate at multiple levels, including individual vulnerability, health-system performance, and broader social and territorial contexts. The identification of clusters does not imply that place itself is causal, but rather that geographic space may capture the cumulative expression of unmeasured or interacting determinants influencing treatment continuity and outcomes.

The spatial concentration of unfavorable tuberculosis outcomes observed in the peripheral zones of the metropolitan area appears to overlap with regions of higher social marginalization. According to national data from the National Population Council (CONAPO), many AGEBs (basic geostatistical areas) in the outskirts of Guadalajara, Tlaquepaque, and Tonalá exhibit high or very high levels of marginalization [20]. Similar patterns have been documented in other urban settings globally, where peripheral areas are often characterized by greater distances to healthcare facilities, reduced healthcare-seeking behavior, and delays in diagnosis and treatment initiation [21,22]. Moreover, these zones frequently overlap with areas of high social deprivation, lower socioeconomic status, and precarious living conditions—all of which have been associated with worse tuberculosis outcomes [23,24,25].

The temporal variation in treatment outcomes observed in this study coincides with the COVID-19 pandemic period, during which significant disruptions to health services were documented in Mexico and globally [2,26]. Although the study did not explicitly aim to measure the impact of the pandemic, the increased odds of unfavorable outcomes in 2021 and 2022, followed by a marked reduction in 2023, are consistent with a period of health system strain and subsequent recovery, potentially influencing diagnosis, follow-up, and treatment continuity. From a spatial perspective, these disruptions may also have altered geographic patterns of care-seeking and service availability, contributing to the transient clustering patterns observed during the early pandemic period [27].

The findings of our study confirm the utility of spatial analysis for understanding variations in tuberculosis treatment outcomes. Other studies conducted in China and Portugal agree with our findings, indicating that the distribution of unfavorable outcomes is not random and that there are distinctive patterns in the area as well as zones of concentration [9]. The geographical concentration of cases with loss to follow-up, treatment failure, or death—even in the absence of significant differences in clinical variables—suggests the possible influence of contextual determinants not captured by conventional records, such as accessibility to the health system, service saturation, or structural social factors [28,29].

Other studies conducted in Mexican context have confirmed this non-random distribution of tuberculosis. A study carried out in Tonalá, Jalisco, found a significant concentration of new pulmonary tuberculosis cases in areas characterized by conditions of marginalization and high population density, suggesting a close relationship between disease transmission and social determinants of health such as overcrowding, poverty, and low educational attainment [30]. These findings are consistent with ours, where we observed a cluster of unfavorable outcomes for the analyzed patients in urban areas, which could be related not only to clinical characteristics but also to contextual factors [24].

In other regions of Mexico, high incidence rates of tuberculosis have been demonstrated, associated with events such as migration, disorganized urbanization, and inequality in medical service coverage [31]. The social backwardness index and its correlation with incidence rates in the southern regions of the country and marginalized urban areas have even been linked [32]. Furthermore, this study not only evidences the clustering of unfavorable outcomes in a defined territory but also underscores the importance of considering the interaction of these geographical variables with individual and clinical factors to develop more effective intervention strategies.

One of the most representative findings of this study was the identification of a significant spatial cluster of patients with unfavorable outcomes in the central area of the study zone, through local autocorrelation analysis. Although these findings should be taken with caution due to possible biases in georeferencing or the lack of additional layers such as marginalization or access to health services, their finding helps to guide the integration of space as a criterion for focusing community interventions. The evidence generated, although exploratory, reinforces the idea of other studies that geographical space may influence disease outcomes through contextual and structural mechanisms and the need to integrate territorial approaches into disease control policies [9,33].

From a spatial analysis perspective, the results derived from the density and Local Moran’s I (LISA) analyses offer critical insights beyond a mere visualization of unfavorable tuberculosis treatment outcomes. These spatial statistical methods enable the identification of significant clusters—such as high-high and low-low groupings—as well as spatial outliers (high-low or low-high), pointing to non-random patterns that might be shaped by structural and contextual factors [14]. In our study, the observed clusters of unfavorable outcomes were concentrated in peripheral zones of the city, suggesting possible spatial segregation and the compounded impact of geographic marginalization.

Our findings reinforce the notion that space is not a neutral backdrop for health phenomena but rather an active dimension that co-produces disease outcomes, as proposed by spatial epidemiology and sociospatial theories of health [19,34]. Areas with high-density and statistically significant clustering likely reflect cumulative disadvantage, including poor access to healthcare services, infrastructure deficits, and social vulnerability. These findings underscore the importance of incorporating place-based variables into the understanding of health inequalities and designing geographically targeted interventions to mitigate spatial health disparities [34].

From an applied public health perspective, the integration of spatial epidemiological tools into routine tuberculosis surveillance systems may support the early identification of geographic areas with a disproportionate burden of unfavorable treatment outcomes. Even when used in an exploratory manner, such analyses can inform prioritization of outreach activities, strengthening of treatment support strategies, and more efficient allocation of resources in complex urban settings served by tertiary-level referral hospitals. Importantly, spatial findings should be interpreted alongside clinical and programmatic data, serving as a complementary decision-support tool rather than a standalone basis for causal inference.

An important consideration when interpreting the spatial clustering observed in this study is the potential influence of referral and catchment area bias. The study population was derived from a tertiary-level referral hospital, which serves as a regional center for the management of more severe, complicated, or treatment-refractory tuberculosis cases. As a result, the geographic distribution of cases—and particularly of unfavorable treatment outcomes—may partially reflect established referral pathways, hospital catchment areas, and patterns of health service utilization rather than community-level risk alone. Spatial hotspots identified in this analysis should therefore not be interpreted solely as indicators of underlying territorial vulnerability, but also as areas contributing disproportionately to the hospital’s clinical workload and referral demand. Nonetheless, from a public health and health systems perspective, these clusters remain highly relevant, as they highlight geographic zones where delayed diagnosis, advanced disease at presentation, or barriers to continuity of care may converge with structural determinants, especially during periods of health system disruption such as the COVID-19 pandemic.

The exclusion of patients with drug-resistant tuberculosis and those who did not initiate treatment may have influenced the observed distribution of unfavorable outcomes and their spatial patterns. Drug-resistant cases often represent more severe or complex clinical presentations and may cluster geographically due to referral pathways or access to specialized services [35]. Their exclusion may therefore lead to an underestimation of the overall burden of unfavorable outcomes in certain areas.

Similarly, individuals who did not initiate treatment may reflect barriers to access, late diagnosis, or social vulnerability, which could also exhibit spatial clustering [36]. Consequently, the spatial patterns identified in this study should be interpreted as reflecting treatment outcomes among patients with drug-susceptible tuberculosis who successfully entered care at a tertiary-level hospital, rather than the full spectrum of tuberculosis cases in the community.

This study presents some limitations that should be acknowledged. Although the sample size is considerable and allows for a robust approximation to spatial analysis, the data come from a captive population attended at a tertiary care unit, which limits the generalization of the findings to other levels of care or non-institutionalized populations. Likewise, although an apparently homogeneous spatial distribution was identified throughout the study area, the concentration of unfavorable outcomes in specific areas may be due, in part, to the natural area of influence of the hospital, which introduces a possible geographical capture bias. Furthermore, the level of analysis is exploratory and not inferential, so additional studies are required to delve deeper into the socio-environmental, structural, and access to services factors that could be influencing the observed distribution. Despite these limitations, the use of spatial tools offers a complementary perspective to clinical and epidemiological analysis and can be fundamental for guiding more focused territorial interventions.

## 5. Conclusions

The findings presented here emphasize that a comprehensive understanding of tuberculosis treatment outcomes requires the integration of spatial epidemiological methods, particularly in identifying areas where patients face heightened risks of unfavorable outcomes.

Our findings advocate for the systematic integration of geospatial analysis into TB surveillance systems, enabling public health authorities to prioritize intervention zones and optimize resource allocation. Strengthening retention in care and improving treatment success rates requires recognizing that place matters: where a person lives continues to influence their ability to access timely diagnosis, remain in treatment, and ultimately survive TB.

Incorporating spatial intelligence into clinical and programmatic decision-making should be viewed as a complementary, exploratory approach that enhances—but does not replace—traditional epidemiological analyses, offering actionable insights for tuberculosis control in urban environments characterized by social vulnerability and health system disruption.

## Figures and Tables

**Table 1:** Sociodemographic and clinical characteristics of the study population.

Variable	Category	Unfavorable Outcome (n = 145) *	Successful Outcome (n = 650)	Total (n = 795)	*p*
Age (IQR)		41 (21)	39 (25)		0.446
Sex	Female	28	197	225	0.008
	Male	117	453	570	
Age group	0–19	7	47	54	0.582
	20–49	92	401	493	
	>50	46	202	248	
Education	Primary	32	85	117	0.023
	Secondary	49	207	256	
	High school	38	194	232	
	No education	20	103	123	
	Higher education	6	61	67	
Employment	Unemployed or no formal occupation	73	232	305	< 0.001
	Homemakers or children	14	113	127	
	Sales or commercial work	5	33	38	
	Manual labor (e.g., carpentry, plumbing)	0	20	20	
	Agricultural/livestock/forestry	3	8	11	
	Professional/specialized fields	11	105	116	
	Other unique professions	39	139	178	
Site of TB disease	Pulmonary	105	385	490	0.004
	Extrapulmonary lymphatic	6	94	100	
	Extrapulmonary pleural	4	14	18	
	Extrapulmonary abdominal	5	39	44	
	Extrapulmonary bone	0	2	2	
	Disseminated extrapulmonary	9	44	53	
	Extrapulmonary CNS	13	33	46	
	Extrapulmonary urinary	0	7	7	
	Other locations	2	26	28	
Living with HIV	No	97	462	559	0.294
	Yes	48	186	234	
Diabetes mellitus	No	135	583	718	0.404
	Yes	10	67	77	
Immunosuppression (not related to HIV infection)	No	123	589	712	0.034
	Yes	22	60	82	
BCG vaccination history	No	30	160	190	0.316
	Yes	115	490	605	
Year of diagnosis	2020	16	99	115	<0.001
	2021	51	148	199	
	2022	77	167	244	
	2023	18	218	236	

* unfavorable outcome includes treatment failure, loss to follow-up, and death (TB-related or unrelated). IQR = Interquartile Range; TB = Tuberculosis; HIV = Human Immunodeficiency Virus; BCG = Bacillus Calmette–Guérin.

**Table 2:** Univariate and multivariate logistic regression analyses.

Variable	Category	Crude OR (95% CI)	*p*	AOR (95% CI)	*p*
**Sex**	Female	Ref			
	Male	1.68 (1.05–2.7)	0.031	1.45 (0.86–2.45)	0.084
**Age group**	0–19	0.76 (0.3–1.93)	0.567	0.66(0.25–1.78)	0.201
	20–49	0.94 (0.62–1.43)	0.775	0.85 (0.52–1.37)	0.251
	>50	Ref			
**Education**	No formal education	1.7 (1.02–2.84)	0.042	1.46 (0.82-2.58)	0.099
	Basic	Ref			
	Middle school or higher	0.61 (0.36–1.05)	0.075	0.68 (0.38–1.21)	0.094
**Employment**	Unemployed or no formal occupation	1.74 (1.17–2.58)	0.006	1.18 (0.75–1.86)	0.232
	Employed	Ref			
**Site of TB disease**	Pulmonary	1.75 (1.13–2.69)	0.012	2.05 (1.27–3.32)	0.001
	Extrapulmonary forms	Ref			
**Living with HIV**	No	Ref			
	Yes	1.19 (0.78–1.82)	0.408	1.44 (0.86–2.41)	0.083
**Year of diagnosis**	2020	Ref			
	2021	2.33 (1.21–4.48)	0.012	2.23 (1.14–4.36)	0.009
	2022	2.44 (1.29–4.62)	0.006	2.27 (1.16–4.3)	0.008
	2023	0.03 (0–0.23)	<0.001	0.03 (0.01–0.20)	<0.001

Footnote: unfavorable outcome was defined as treatment failure, loss to follow-up, or death (TB-related or unrelated). AOR: Adjusted Odds Ratio; CI: Confidence Interval; TB: Tuberculosis; HIV: Human Immunodeficiency Virus.

## Data Availability

The data that support the findings of this study are available from the corresponding author upon reasonable request.
