# Correction: Del Moral Trinidad et al. Spatial Epidemiological Approach to Tuberculosis Treatment Outcomes in a Tertiary-Level Hospital: A Retrospective Analysis. *Trop. Med. Infect. Dis.* 2026, *11*, 57

**DOI:** 10.3390/tropicalmed11030074

**Published:** 2026-03-06

**Authors:** Luis Eduardo Del Moral Trinidad, Gilberto Silva Bañuelos, Esteban Gonzalez-Diaz, Melva Guadalupe Herrera Godina

**Affiliations:** 1 Doctorado en Ciencias de la Salud Pública, Centro Universitario de Ciencias de la Salud, Universidad de Guadalajara, Guadalajara 44430, Mexico; l.eduardo.trinidad@outlook.com; 2 Departamento de Salud Pública, Centro Universitario de Ciencias de la Salud, Universidad de Guadalajara, Guadalajara 44430, Mexico; gil.saludpublica@gmail.com; 3 Unidad de Medicina Preventiva y Vigilancia Epidemiológica, Hospital Civil de Guadalajara “Fray Antonio Alcalde”, Guadalajara 44280, Mexico; esteban.gdiaz@academicos.udg.mx; 4 Instituto de Patología Infecciosa y Experimental “Francisco Ruiz Sánchez”, Centro Universitario de Ciencias de la Salud, Universidad de Guadalajara, Guadalajara 44430, Mexico

In the published publication [1], there was an error and missing details in affiliation 1. The original affiliation 1 was Department of Public Health, University of Guadalajara, Guadalajara 44430, Mexico. In addition, the affiliation numbers were wrong.

The new authorship is updated as:

Luis Eduardo Del Moral Trinidad ^1^, Gilberto Silva Bañuelos ^2^, Esteban Gonzalez-Diaz ^3,4^ and Melva Guadalupe Herrera Godina ^2,^*

^1^  Doctorado en Ciencias de la Salud Pública, Centro Universitario de Ciencias de la Salud, Universidad de Guadalajara, Guadalajara 44430, Mexico; l.eduardo.trinidad@outlook.com
^2^  Departamento de Salud Pública, Centro Universitario de Ciencias de la Salud, Universidad de Guadalajara, Guadalajara 44430, Mexico; gil.saludpublica@gmail.com
^3^  Unidad de Medicina Preventiva y Vigilancia Epidemiológica, Hospital Civil de Guadalajara “Fray Antonio Alcalde”, Guadalajara 44280, Mexico; esteban.gdiaz@academicos.udg.mx
^4^  Instituto de Patología Infecciosa y Experimental “Francisco Ruiz Sánchez”, Centro Universitario de Ciencias de la Salud, Universidad de Guadalajara, Guadalajara 44430, Mexico

* Correspondence: melva.herrera@academicos.udg.mx; Tel.: +52-3-312-550-196

The authors state that the scientific conclusions are unaffected. This correction was approved by the Academic Editor. The original publication has also been updated.
